# Multiple *PIK3CA* mutation clonality correlates with outcomes in taselisib + fulvestrant-treated ER+/HER2–, *PIK3CA*-mutated breast cancers

**DOI:** 10.1186/s13073-023-01181-8

**Published:** 2023-04-26

**Authors:** Katherine E. Hutchinson, Jessica W. Chen, Heidi M. Savage, Thomas J. Stout, Frauke Schimmoller, Javier Cortés, Susan Dent, Nadia Harbeck, William Jacot, Ian Krop, Sally E. Trabucco, Smruthy Sivakumar, Ethan S. Sokol, Timothy R. Wilson

**Affiliations:** 1 Oncology Biomarker Development, Genentech, Inc., 1 DNA Way, South San Francisco, CA 94080 USA; 2 Product Development Oncology, Genentech, Inc., South San Francisco, CA USA; 3 International Breast Cancer Center (IBCC), Pangaea Oncology, Quironsalud Group, Madrid & Barcelona, Spain; 4 Department of Medicine, Faculty of Biomedical and Health Sciences, Universidad Europea de Madrid, Madrid, Spain; 5 Duke Cancer Institute, Duke University, Durham, NC USA; 6 Breast Center, Department Gynecology and Obstetrics and Comprehensive Cancer Center (CCC) Munich, Ludwig-Maximilians-University (LMU) Hospital, Munich, Germany; 7 Institut du Cancer de Montpellier (ICM) Val d’Aurelle, Montpellier University, INSERM U1194, Montpellier, France; 8 Yale Cancer Center, New Haven, CT USA; 9 Foundation Medicine, Inc, Cambridge, MA USA

**Keywords:** *PIK3CA*, Double *PIK3CA* mutation, Clonal, Taselisib, PI3K inhibitor, Breast cancer, ctDNA, PI3K signaling

## Abstract

**Background:**

Mutations in the p110α catalytic subunit of phosphatidylinositol 3-kinase (PI3K), encoded by the *PIK3CA* gene, cause dysregulation of the PI3K pathway in 35–40% of patients with HR+/HER2– breast cancer. Preclinically, cancer cells harboring double or multiple *PIK3CA* mutations (mut) elicit hyperactivation of the PI3K pathway leading to enhanced sensitivity to p110α inhibitors.

**Methods:**

To understand the role of multiple *PIK3CA*mut in predicting response to p110α inhibition, we estimated the clonality of multiple *PIK3CA*mut in circulating tumor DNA (ctDNA) from patients with HR+/HER2– metastatic breast cancer enrolled to a prospectively registered clinical trial of fulvestrant ± taselisib, and analyzed the subgroups against co-altered genes, pathways, and outcomes.

**Results:**

ctDNA samples with clonal multiple *PIK3CA*mut had fewer co-alterations in receptor tyrosine kinase (RTK) or non-*PIK3CA* PI3K pathway genes compared to samples with subclonal multiple *PIK3CA*mut indicating a strong reliance on the PI3K pathway. This was validated in an independent cohort of breast cancer tumor specimens that underwent comprehensive genomic profiling. Furthermore, patients whose ctDNA harbored clonal multiple *PIK3CA*mut exhibited a significantly higher response rate and longer progression-free survival vs subclonal multiple *PIK3CA*mut.

**Conclusions:**

Our study establishes clonal multiple *PIK3CA*mut as an important molecular determinant of response to p110α inhibition and provides rationale for further clinical investigation of p110α inhibitors alone or with rationally-selected therapies in breast cancer and potentially other solid tumor types.

**Supplementary Information:**

The online version contains supplementary material available at 10.1186/s13073-023-01181-8.

## Background

The phosphatidylinositol 3-kinase (PI3K) pathway regulates pro-survival and pro-growth cellular signaling, but is frequently dysregulated in solid tumors [1–4]. In particular, approximately 40% of hormone-receptor positive, human epidermal growth factor receptor 2-negative (HR+/HER2–) breast cancers (BCs) exhibit aberrant activation of the PI3K pathway through gain-of-function mutations in the α isoform of PI3K. PI3Kα is an obligate complex of a regulatory p85 domain and the catalytic α isoform phosphatidylinositol-4,5-bisphosphate 3-kinase (p110α), which is encoded by the *PIK3CA* gene [2, 5, 6]. Although *PIK3CA* mutations (*PIK3CA*mut) can occur throughout the gene, the majority of *PIK3CA*mut occur in two “hotspot” regions of p110α: the helical domain (primarily amino acids E542 and E545); and the kinase domain (primarily amino acid H1047) [2, 7, 8].

*PIK3CA*mut have long been a target of clinical investigation as a likely predictive biomarker of response to PI3K pathway inhibitors. Early pan-PI3K isoform inhibitors and dual inhibitors of PI3K and mTOR such as buparlisib, pictilisib, and apitolisib, were unable to fully validate *PIK3CA*mut as a predictive biomarker due to dose-limiting toxicities [9–12]. However, more recent inhibitors, such as the p110β-sparing inhibitor, taselisib, and the p110α-selective inhibitor, alpelisib, have demonstrated statistically significant improvements in progression free survival in phase III studies [13, 14]. Alpelisib was approved by the Food and Drug Administration (FDA) in 2019 for the treatment of patients with HR+/HER2– *PIK3CA*mut advanced or metastatic breast cancer in combination with fulvestrant, an estrogen receptor degrader, thus validating *PIK3CA*mut as a predictive biomarker of response to PI3K pathway inhibition.

Despite these successes, there remains an unmet need to develop PI3K inhibitors with improved isoform specificity and anti-cancer efficacy. To guide this development, further refinement of our understanding of *PIK3CA*mut and subcategories of *PIK3CA*mut as predictive biomarkers for patients with cancer is necessary. Building from early observations of exceptional responders in an alpelisib phase I trial [15] Vasan, et al., determined that co-occurring *PIK3CA*mut within the same tumor (i.e., “double *PIK3CA*mut”), especially those occurring together on the same allele (*in cis*), were the likely source of these exceptional responses [16]. Through in silico and preclinical modeling efforts, the authors demonstrated that double *PIK3CA*mut *in cis* elicit hyperactivation of the PI3K pathway through enhanced membrane binding that resulted in both increased cellular proliferation and sensitivity to p110α-selective inhibitors, such as alpelisib and GDC-0077 (inavolisib) [16].

Taselisib was investigated in combination with fulvestrant in the phase III clinical trial, SANDPIPER, for patients with estrogen-receptor positive (ER+), HER2– locally advanced or metastatic breast cancer (NCT02340221) [14]. The study met its primary endpoint of improved progression-free survival (PFS) with taselisib + fulvestrant over placebo + fulvestrant albeit with modest clinical activity (7.4 vs 5.4 months, respectively; hazard ratio = 0.70) [14]. SANDPIPER is the largest randomized clinical trial of a PI3K inhibitor in patients with *PIK3CA*mut tumors. Of note, when categorizing patients’ tumors as *PIK3CA*mut by baseline circulating tumor DNA (ctDNA), a larger treatment effect was observed compared to outcomes based on tissue *PIK3CA*mut positivity (irrespective of ctDNA-based status), especially in patients with more than one *PIK3CA*mut in their ctDNA compared to those with a single *PIK3CA*mut in their ctDNA [14, 16].

To further investigate this observation, we employed an in silico﻿ methodology to infer the clonality of single and multiple *PIK3CA*mut identified in plasma-derived ctDNA from participants enrolled to SANDPIPER, and analyzed the resultant subgroups against co-altered genes and pathways, as well as against clinical outcomes. To validate our findings, we similarly interrogated a large genomic database of patient breast cancer tumors that underwent comprehensive genomic profiling [17, 18]. Herein, we establish the clonal aspects of single and multiple *PIK3CA*mut, enhance our understanding of how multiple *PIK3CA*mut impact ability of other cellular signaling pathways to influence anti-tumor responses of patients with HR+/HER2– metastatic BC to PI3K inhibition, and provide justification for new rational therapeutic combination approaches.

## Methods

### SANDPIPER clinical trial

Between 9 April 2015 and 4 September 2017, 631 postmenopausal patients aged 18 years or older with ER+, HER2– locally advanced or metastatic breast cancer and who experienced disease recurrence/progression during or after aromatase inhibitor therapy were enrolled to the prospectively registered phase III clinical trial, SANDPIPER (NCT02340221) in a 2:1 fashion for treatment with taselisib (GDC-0032) + fulvestrant (*n* = 417) versus placebo + fulvestrant (*n* = 214) [14]. Based on centralized testing of tumor tissue with the cobas^®^ *PIK3CA* Mutation Test, patients with *PIK3CA*mut tumors were randomized separately from those in which no *PIK3CA*mut was detected (NMD). Stratification factors included visceral disease, endocrine sensitivity, and geographic region. Notable exclusion criteria include HER2-positive (HER2+) disease, prior treatment with fulvestrant, and prior treatment with a PI3K, mTOR, or AKT inhibitor. For details regarding ethical consent to participate, please refer to the Declarations section of this manuscript. In brief, the SANDPIPER study met its primary endpoint of improved progression-free survival (PFS) with taselisib + fulvestrant over placebo + fulvestrant in patients with *PIK3CA*mut tumors (7.4 vs 5.4 months, respectively; hazard ratio = 0.70). The secondary endpoints, including objective response rate (ORR), clinical benefit rate (CBR), duration of objective response (DoR), and PFS per blinded independent central review (BICR-PFS), showed consistent improvement with taselisib + fulvestrant over placebo + fulvestrant in the *PIK3CA*mut population. Overall survival (OS) data were immature at the time of the original report [14].

### Plasma ctDNA collection and comprehensive genomic profiling (CGP)

ctDNA was isolated as previously described [19] from plasma collected immediately prior to treatment (i.e., at baseline) from 631 participants enrolled to the SANDPIPER clinical trial [14]. CGP of baseline plasma samples from 598 participants was performed with the FoundationOne^®^Liquid (F1L) assay in a Clinical Laboratory Improvement Amendments (CLIA)-certified, College of American Pathologists (CAP)-accredited reference laboratory (Foundation Medicine, Inc., Cambridge, MA, USA). F1L uses hybrid-capture, adapter ligation-based libraries to identify genomic alterations (base substitutions, small insertions and deletions, copy number alterations, and select rearrangements/fusion events) in 70 cancer-related genes. Comprehensive details on the F1L platform, sequencing, and mutation calling methodologies were previously described [19]. Of the 598 plasma samples that underwent CGP, 508 of these samples were successfully sequenced, having passed quality control parameters for DNA concentrations, library preparation, tumor purity, and target region coverage.

**Fig. 1:**
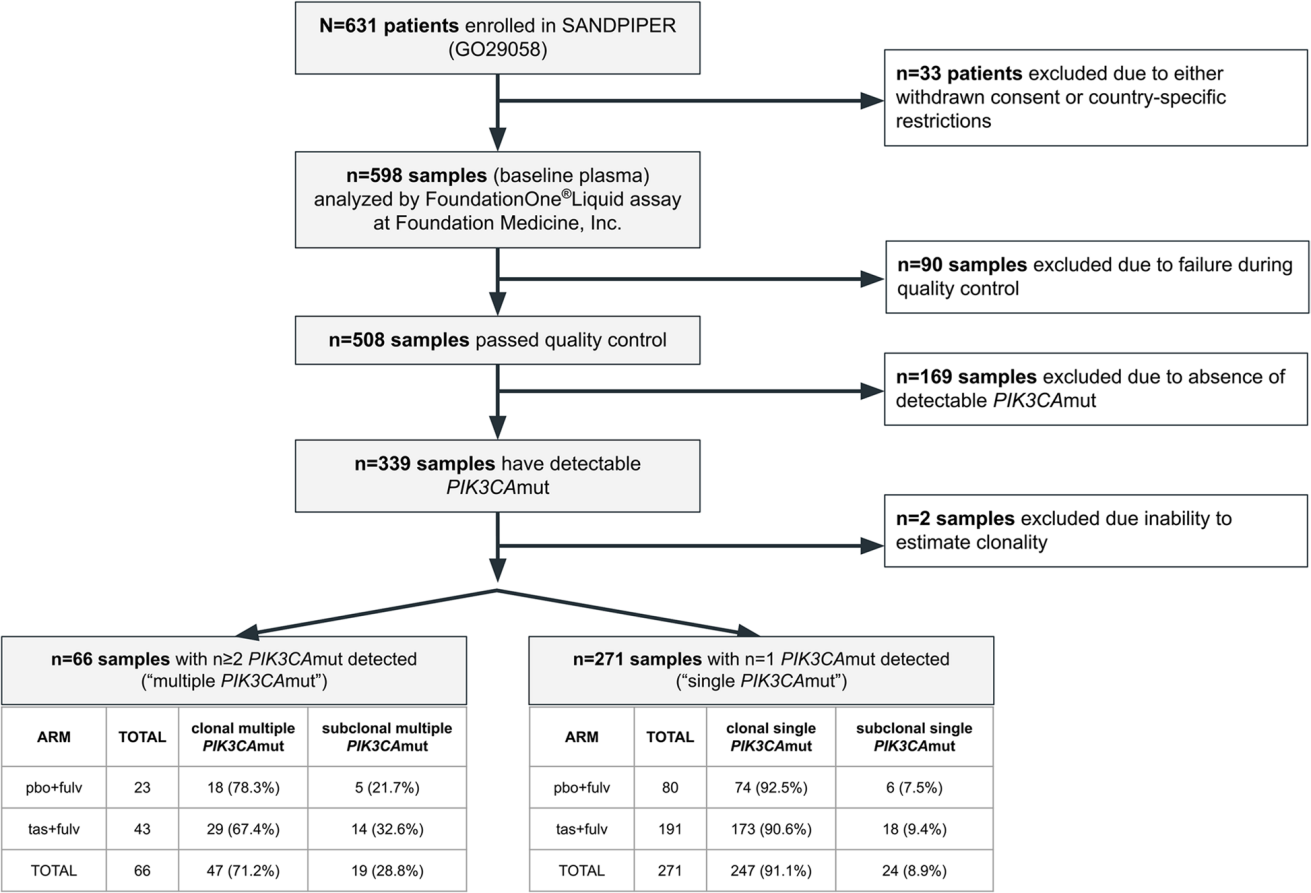
Flow diagram of baseline ctDNA samples analyzed and estimated clonality collected from SANDPIPER participants. *PIK3CA*mut is defined as a single nucleotide variant in the *PIK3CA* gene that is predicted to be of known or likely oncogenic significance

Three hundred thirty-nine of these baseline plasma ctDNA samples harbored one or more pathogenic *PIK3CA* single-nucleotide variant(s) (*PIK3CA*mut), defined as a variant of known or likely oncogenic significance, as described by Clark, *et al. *[19]. Two samples were excluded from subsequent analysis due to the inability to estimate clonality (see Methods subsection “*PIK3CA*mut clonality estimation” below for more details). The resultant 337 *PIK3CA*mut patient samples represent the final study population for the downstream analyses described herein, sub-categorized based on the number of *PIK3CA*mut detected in baseline ctDNA and the estimated clonality of those *PIK3CA*mut (see again, Methods subsection “*PIK3CA*mut clonality estimation” below). Samples with one detectable *PIK3CA*mut were classified as ‘single *PIK3CA*mut’ (*n* = 271) and those with ≥ 2 detectable *PIK3CA*mut were classified as ‘multiple *PIK3CA*mut’ (*n* = 66). See Fig. 1 for details.

### Independent breast cancer dataset

Comprehensive genomic profiling (CGP) was performed on formalin-fixed paraffin-embedded (FFPE) breast tumor samples submitted to Foundation Medicine, Inc. (Cambridge, MA, USA) during the course of routine clinical care through March 31, 2021. Profiling was done for all classes of alterations in at least 324 genes using the FoundationOne^®^ (F1) or FoundationOne^®^CDx (F1CDx) assay in a CLIA-certified, CAP-accredited laboratory (Foundation Medicine, Inc., Cambridge, MA, USA) [20]. For the analyses herein, samples were filtered from adult patients (≥ 18 years old) and for samples that harbored ≥ 2 pathogenic single-nucleotide variants in the *PIK3CA* gene. Processing of sequence data and identification of different classes of genomic alterations were performed as previously described [17, 18]. The variant post processing and harmonization process is maintained across the ctDNA and tissue-based assays.

### *PIK3CA*mut clonality estimation

For the SANDPIPER cohort, the clonality of each *PIK3CA*mut was estimated based on the alteration allele fraction and the estimated ctDNA fraction. The ctDNA fraction was estimated based on tumor aneuploidy or maximum somatic allele fraction, as described previously [21–23]. Briefly, when there is clear evidence of aneuploidy (~ 10% tumor shed), an aneuploidy-based estimate of tumor fraction is used. In cases without clear aneuploidy, a maximum somatic allele fraction (MSAF), the highest variant allele fraction among all non-germline variants and after excluding specific clonal hematopoiesis-associated alterations, was used. Clonal fraction (i.e., estimated clonality) of a variant was calculated as the ratio of the variant allele fraction (VAF) to the sample estimated ctDNA fraction. Variants with a clonal fraction of ≥ 0.25 were classified as clonal; variants with a clonal fraction < 0.25 were classified as subclonal. This cutoff was chosen as it represents the clonal fraction where a heterozygous single copy alteration would be identified in a majority of diploid tumor cells. These clonality estimates do not take into account the zygosity and loci copy number estimates. In cases of variants on an amplified allele, this can result in overestimation of tumor fraction or variant clonal fraction. Herein, for samples with a single *PIK3CA*mut, a 'clonal single *PIK3CA*mut' is defined as a mutation for which the clonal fraction is ≥ 0.25; a 'subclonal single *PIK3CA*mut' is defined as a mutation for which the clonal fraction is < 0.25. For samples with multiple *PIK3CA*mut detected, the *PIK3CA* clonality category is defined as follows: ‘clonal multiple *PIK3CA*mut’: samples for which at least two (≥ 2) clonal *PIK3CA*mut were detected; ‘subclonal multiple *PIK3CA*mut’: samples for which at most one (≤ 1) clonal *PIK3CA*mut was detected. These classifications and nomenclature are also succinctly summarized in Additional file 1: Table S1. Two samples for which the clonality of the *PIK3CA*mut could not be estimated and therefore could not be classified, were excluded from the analysis (Fig. 1).

In the independent dataset of multiple *PIK3CA*mut breast cancer tissue samples, we utilized estimates of total and mutated copy number from the somatic-germline-zygosity (SGZ) algorithm [24] to calculate the clonality [25]. For each predicted somatic alteration observed in a sample, a tumor fraction was estimated using the following formula: 2AF / (mc – AF(wc + mc-2)), where AF indicates the variant allele fraction, and mc and wc indicate the mutated copies and wild-type copies, respectively. The tumor fraction of the sample was then calculated as the maximum estimated tumor fraction from all the somatic mutations in a sample. Clonal fraction of each mutation was then obtained as the ratio of the AF and sample estimated tumor fraction, with ≥ 50% considered clonal. *PIK3CA* clonality categories were derived similarly to the SANDPIPER cohort.

### Signaling pathway cluster analysis

Signaling pathway alteration status was defined by the detection of at least one pathogenic alteration (i.e., base substitution, insertion and deletion, copy number alteration, or rearrangement of known or likely oncogenic significance) in any gene from the associated pathway gene list in Additional file 1: Table S4. Of note, *PIK3CA* was removed from the PI3K pathway gene list for the indicated pathway-level analyses; all participants in the analysis cohort harbored a pathogenic *PIK3CA* mutation. The gene list used for the signaling pathway level analysis for both the SANDPIPER dataset (F1L sequencing of baseline ctDNA) and the independent dataset from Foundation Medicine, Inc. (F1 or F1CDx sequencing of tumor tissue) was guided by and restricted to the genes included in the F1L gene panel.

### Analysis of patient response to PI3K inhibition

Patients enrolled in SANDPIPER were stratified into *PIK3CA*mut subgroups (see Results) against which objective response rate (ORR), represented by the number of patients who responded to treatment (defined by best objective response per investigator assessment, including CR and PR) divided by the total number of patients in each subgroup, and progression-free survival (PFS), defined as time from randomization to first evidence of disease progression as determined by the investigator using Response Evaluation Criteria in Solid Tumors version 1.1 (RECIST v1.1) or death from any cause, were assessed. Differences in the ORR among patients in the *PIK3CA*mut subgroups were compared (*p*-value) using a stratified exact conditional test. Differences in the PFS among patients in the *PIK3CA*mut subgroups were compared using the Kaplan–Meier method wherein associated hazard ratios and *p*-values were obtained from Cox proportional hazards regression models and log-rank test, respectively.

### Statistical analyses and software

Statistics, computation, and plotting were performed using Python 2.7 and R 3.6.1. Because analyses were exploratory, *p*-values were not adjusted for multiple testing. A *p*-value ≤ 0.05 was considered significant for the analyses herein.

## Results

### Clonality estimation of multiple *PIK3CA*mut in ctDNA

Of the 631 patients enrolled to SANDPIPER, 339 baseline ctDNA samples were successfully sequenced and found to harbor at least one detectable *PIK3CA*mut (Fig. 1). After excluding two samples for the inability to estimate clonality, of these, one *PIK3CA*mut was detected in 271 samples and are herein termed single *PIK3CA*mut; ≥ 2 *PIK3CA*mut were detected in 66 samples and are herein termed multiple *PIK3CA*mut. Samples were further assigned to a *PIK3CA*mut clonality category of clonal or subclonal based on a clonality estimation algorithm. Refer to the Methods and Additional file 1: Table S1 for specific details.

Reflective of the 2:1 randomization schema for SANDPIPER, of the 66 samples in which multiple *PIK3CA*mut were identified, 23 (34.8%) were from patients treated with placebo + fulvestrant (pbo + fulv) and 43 (65.2%) were from patients treated with taselisib + fulvestrant (tas + fulv). Upon clonality estimation, the majority of the multiple *PIK3CA*mut were categorized as clonal [47 of 66 (71.2%); 18 of 23 (78.3%) in the pbo + fulv arm; 29 of 43 (67.4%) in the tas + fulv arm] and a lower number were categorized as subclonal [19 of 66 (28.8%); 5 of 23 (21.7%) in the pbo + fulv arm; 14 of 43 (32.6%) in the tas + fulv arm] (Fig. 1). Within the subclonal multiple *PIK3CA*mut cohort, the majority (78.9%; *n* = 15 of 19) harbored one *PIK3CA*mut with a clonality estimate of clonal and at least one *PIK3CA*mut with a clonality estimate of subclonal, and a minority (21.1%; *n* = 4 of 19) harbored only *PIK3CA*mut with a clonality estimate of subclonal. Similarly, of the 271 samples in which a single *PIK3CA*mut was identified, the majority were classified as clonal [247 of 271 (91.1%); 74 of 80 (92.5%) in the pbo + fulv arm; 173 of 191 (90.6%) in the tas + fulv arm] (Fig. 1). Twenty-four samples with a single *PIK3CA*mut were classified as subclonal [24 of 271 (8.9%); 6 of 80 (7.5%) in the pbo + fulv arm; 18 of 191 (9.4%) in the tas + fulv arm] (Fig. 1). The specific *PIK3CA*mut and combinations of *PIK3CA*mut (e.g., H1047R + E726K, E545K + P539R, etc.) identified, clonality categorizations, and distribution between study treatment arms can be found in Additional file 1: Tables S2 & S3.

### Clonal multiple *PIK3CA*mut exhibit fewer RTK co-alterations

**Fig. 2:**
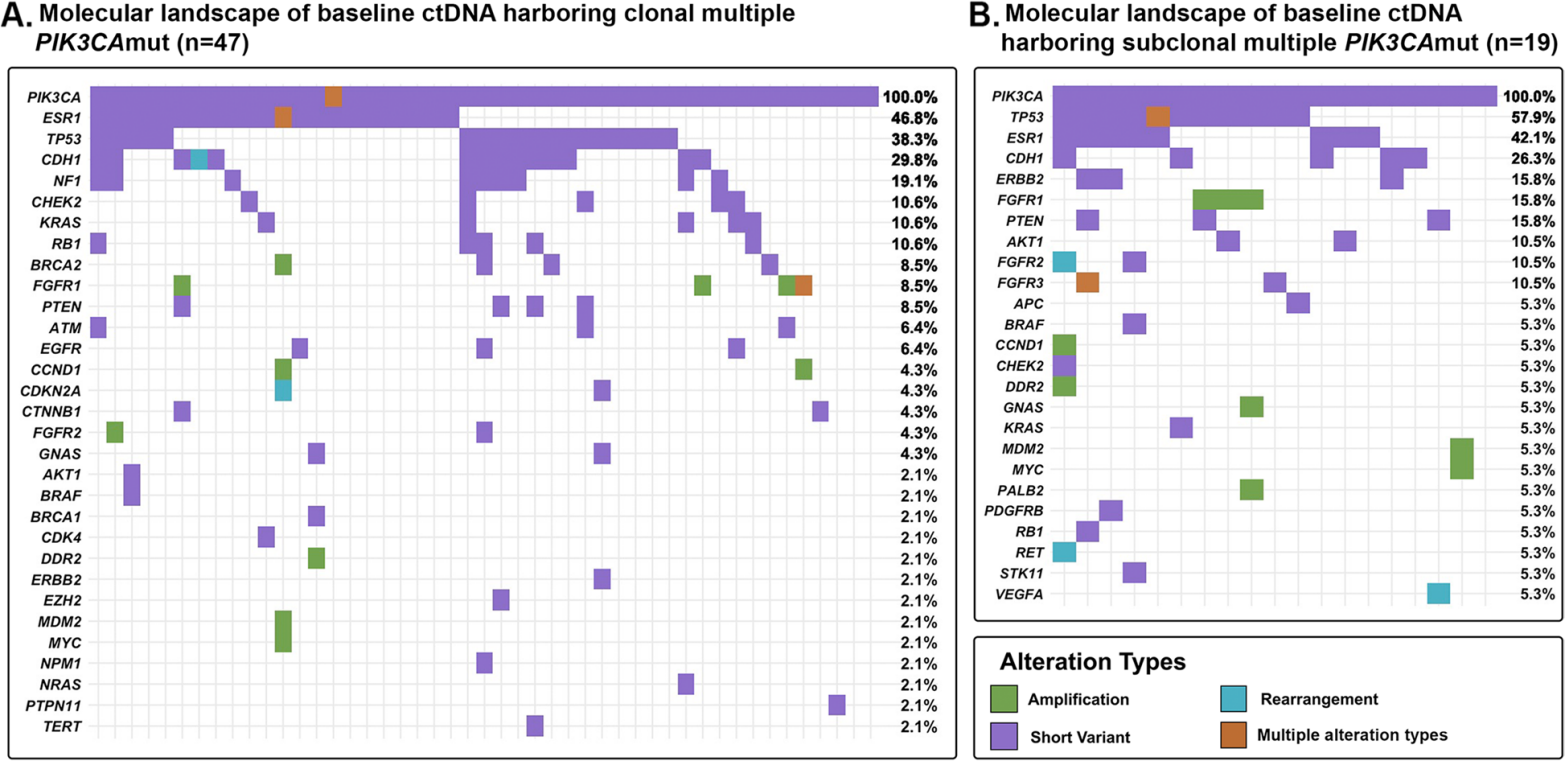
Gene alteration rates in baseline ctDNA are not different between clonal vs subclonal multiple *PIK3CA*mut. Tile plots from FoundationOne^®^Liquid (F1L) sequencing of baseline ctDNA from SANDPIPER participants exhibit the somatic alterations co-occurrent with samples categorized as (**A**) clonal multiple *PIK3CA*mut or (**B**) subclonal multiple *PIK3CA*mut. Samples are represented in the columns, whereas genes are represented in the rows. No statistically significant difference was observed in the individual gene alteration rates between these *PIK3CA*mut clonality subgroups (*p*-value > 0.05; Fisher’s Exact Test). Orange boxes represent samples with multiple alteration types identified in one gene

Using clonality estimation, we sought to determine whether the mutational landscape differs between breast cancers harboring clonal multiple *PIK3CA*mut compared to BCs harboring subclonal multiple *PIK3CA*mut regardless of treatment arm. As illustrated by tile plots, a comparison of the alteration rates of individual genes between clonal (Fig. 2A) vs subclonal (Fig. 2B) multiple *PIK3CA*mut samples revealed no statistically significant differences. Among the samples in which only a single *PIK3CA*mut was detected, a statistically lower prevalence of *MDM2* amplifications and *BRCA2* alterations was observed in those categorized as clonal single *PIK3CA*mut compared to those categorized as subclonal single *PIK3CA*mut [*MDM2*: 1.2% (3 of 247 samples) vs 12.5% (3 of 24 samples), *p* = 0.0103; *BRCA2*: 2.4% (6 of 247 samples) vs 12.5% (3 of 24 samples), *p* = 0.0361] (Additional file 1: Fig. S1).

**Fig. 3:**
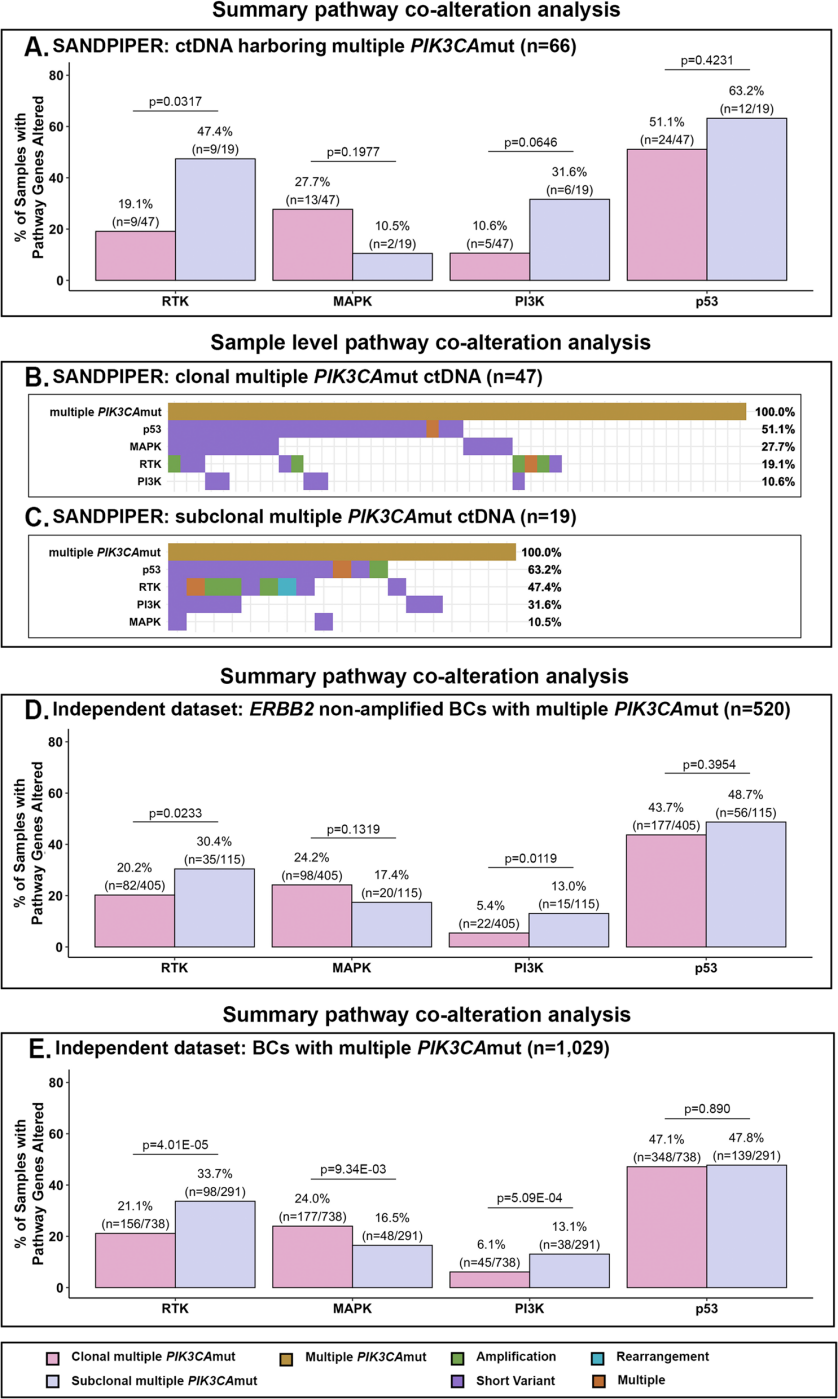
Clonal multiple *PIK3CA*mut samples exhibit fewer RTK and non-*PIK3CA* PI3K pathway gene alterations. **A** Summary diagram of pathway-level alteration rates in baseline ctDNA from SANDPIPER participants shows a significantly lower prevalence of alterations in genes associated with receptor tyrosine kinase (RTK) signaling in those samples with clonal vs subclonal multiple *PIK3CA*mut (*p* = 0.0317); (**B** & **C**), tile plot of sample-level pathway co-alteration analysis. Summary diagrams of pathway-level alteration rates in a larger dataset of (**D**) *ERBB2* non-amplified metastatic breast cancer (BC) tumor tissue samples and **(E)** any BC tumor tissue samples from the Foundation Medicine database harboring clonal multiple *PIK3CA*mut exhibit a lower prevalence of alterations in RTK-related genes (*p* = 0.0233 and *p* = 4.01 × 10^–5^, respectively) and in non-*PIK3CA* PI3K-pathway genes (*p* = 0.0119 and *p* = 5.09 × 10^–4^, respectively) compared to samples harboring subclonal multiple *PIK3CA*mut. A higher proportion of alterations in MAPK pathway genes was observed in breast cancer samples with clonal multiple *PIK3CA*mut vs those with subclonal multiple *PIK3CA*mut (*p* = 9.34 × 10^–3^) (**E**). *p*-values were obtained from a Fisher’s Exact Test. KEYS: For the summary pathway co-alteration analyses, pink and lavender bars represent samples with clonal or subclonal multiple *PIK3CA*mut, respectively. For alteration type specifications, distinct from “multiple *PIK3CA*mut”, orange boxes represent samples with multiple alteration types identified in one gene

Analysis of these data at the signaling pathway level (see Additional file 1: Table S4 for pathway gene lists), revealed that patients with ctDNA harboring clonal multiple *PIK3CA*mut have a lower prevalence of alterations in genes associated with receptor tyrosine kinase (RTK) signaling in baseline ctDNA samples [9 of 47 samples (19.1%)] compared to subclonal multiple *PIK3CA*mut [9 of 19 samples (47.4%), *p* = 0.032] (Fig. 3A). Cumulative alterations in other analyzed signaling pathways [i.e., the p53, MAPK, or PI3K (excluding *PIK3CA*) pathways] were not statistically different between multiple *PIK3CA*mut clonality groups, despite showing numerical differences. Between treatment groups, no differences were observed in the prevalence of RTK, MAPK, PI3K, or p53 pathway genes altered (Additional file 1: Table S5 and S6; *p*-value > 0.05, Fisher’s Exact Test). Additionally, the prevalences of RTK, MAPK, PI3K, and p53 pathway genes altered within the subclonal multiple *PIK3CA*mut subgroup, wherein one of the *PIK3CA* mutations has a clonality estimate of clonal (*n* = 15), were similar to the prevalences observed in the entire subclonal multiple *PIK3CA*mut cohort (data not shown). Furthermore, pathway-grouped (i.e., RTK, p53, MAPK, or PI3K) alterations were neither co-occurrent nor mutually exclusive of one another in samples categorized to either multiple *PIK3CA*mut clonality group (Fig. 3B-C). Interestingly, the same pathway-level analysis of samples in which only a single *PIK3CA*mut was identified revealed no statistically significant differences in pathway-grouped gene alterations between clonal and subclonal groups (Additional file 1: Fig. S2).

### Independent dataset analysis of clonal *PIK3CA*mut findings

To determine whether our pathway-level findings in the SANDPIPER ctDNA dataset are supported by a second dataset, we interrogated all available breast tumor tissue samples in an independent database for which *PIK3CA*mut and clonality calls could be estimated (see Methods and Additional file 1: Fig. S3). The database consists of real-world genomic data from patients whose tumor tissue was submitted for comprehensive genomic profiling as part of routine clinical care. Within this dataset, 1,029 breast cancer tumor samples harbored multiple *PIK3CA*mut, with a majority estimated as clonal [738 of 1,029 (71.7%)] (Additional file 1: Fig. S3). Consistent with the SANDPIPER cohort, a similar proportion of clonal multiple *PIK3CA*mut [405 of 520 (77.9%)] was observed in the subgroup of breast cancer tissue samples that were *ERBB2* non-amplified and biopsied from a metastatic site of disease (Additional file 1: Fig. S3), which more closely resembles the trial population.

Similar to our observations in the SANDPIPER ctDNA dataset, clonal multiple *PIK3CA*mut samples from the metastatic breast tumor tissue dataset exhibited significantly fewer alterations in genes associated with RTK signaling [82 of 405 samples (20.2%)] compared to samples with subclonal multiple *PIK3CA*mut [35 of 115 samples (30.4%), *p* = 0.0233] (Fig. 3D). In the SANDPIPER ctDNA dataset, we observed a trend of fewer alterations in PI3K (excluding *PIK3CA*) pathway genes in samples with clonal vs subclonal multiple *PIK3CA*mut, however this did not reach statistical significance. In the larger dataset of metastatic tumor tissue from patients with *ERBB2* non-amplified breast cancer, however, this finding was statistically significant [22 of 405 samples (5.4%) vs 15 of 115 samples (13.0%), *p* = 0.0119] (Fig. 3D). Taking a broader look across all breast cancer samples (inclusive of primary and metastatic tumor tissue) in the independent dataset with multiple *PIK3CA*mut samples, a significantly lower proportion of alterations in RTK pathway genes and non-*PIK3CA* PI3K pathway genes was observed in samples categorized as clonal multiple *PIK3CA*mut compared to those categorized as subclonal multiple *PIK3CA*mut [Fig. 3E, RTK: 156 of 738 clonal multiple *PIK3CA*mut samples (21.1%) vs 98 of 291 subclonal multiple *PIK3CA*mut samples (33.7%), *p* = 4.01 × 10^–5^; PI3K: 45 of 738 clonal multiple *PIK3CA*mut samples (6.1%) vs 38 of 291 subclonal multiple *PIK3CA*mut samples (13.1%), *p* = 5.09 × 10^–4^]. Conversely, a significantly higher proportion of alterations in MAPK pathway genes (Additional file 1: Table S4) was observed in breast cancer samples categorized as clonal multiple *PIK3CA*mut compared to those categorized as subclonal multiple *PIK3CA*mut [Fig. 3E, 177 of 738 samples (24.0%) vs 48 of 291 samples (16.5%), *p* = 9.34 × 10^–3^]. Notwithstanding the similarities in the findings from the SANDPIPER baseline ctDNA and the independent breast cancer tumor tissue, differences exist in the timing of the specimen collection and the NGS-based methodologies (e.g., ctDNA- versus tissue-based) that may contribute to the datasets not being contemporary. For example, the baseline plasma samples from the SANDPIPER study were freshly collected during the study screening period prior to initiation of 2L treatment, whereas collection of the metastatic tumor tissue samples was not restricted to the early line metastatic setting.

### Clonal multiple *PIK3CA*mut associate with improved clinical outcomes

**Fig. 4:**
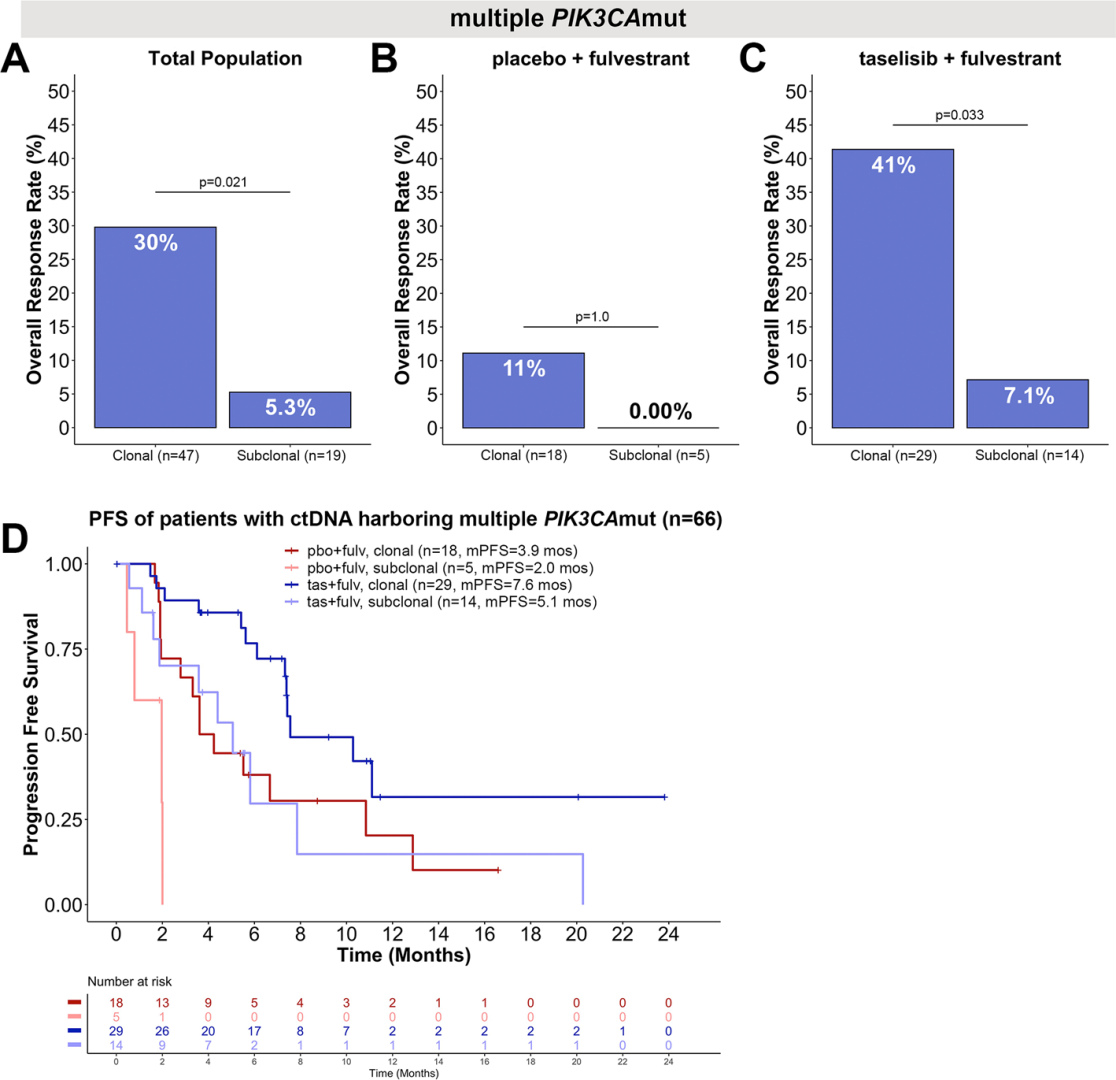
Clonal multiple *PIK3CA*mut status correlates with higher ORR and longer PFS. **A – C** Bar plots of overall objective response rate (ORR) for SANDPIPER study participants show that ORR trends higher or is significantly higher in those whose baseline ctDNA harbored clonal vs subclonal multiple *PIK3CA*mut. **A** Study participants who received either study treatment regimen: clonal [14 responses of 47 participants = 30% ORR (95% CI, 17–45)] vs subclonal [1 response of 19 participants = 5.3% ORR (95% CI, 0.13–26)]; *p* = 0.021. **B** Study participants who received placebo + fulvestrant: clonal [2 responses of 18 participants = 11% ORR (95% CI, 1.4–35)] vs subclonal [0 responses of 5 participants = 0.00% ORR (95% CI, 0.0–52)]; *p* = 1.0. **C** Study participants who received taselisib + fulvestrant: clonal [12 responses of 29 participants = 41% ORR (95% CI, 243.5–61)] vs subclonal [1 response of 14 participants = 7.1% ORR (95% CI, 0.18–34)]; *p* = 0.033. **D** Shown via Kaplan–Meier curves, median progression-free survival (PFS) was longer for those whose corresponding baseline ctDNA harbored clonal multiple *PIK3CA*mut vs subclonal multiple *PIK3CA*mut and were treated with either placebo + fulvestrant [median PFS (mPFS) = 3.9 vs 2.0 months, respectively; Hazard Ratio (HR) = 0.19 (95% CI, 0.051–0.73), *p* = 0.025] or with taselisib + fulvestrant [mPFS = 7.6 vs 5.1 months, respectively; HR = 0.37 (95% CI, 0.16–0.87), *p* = 0.027]. See Methods on details for calculations and statistics of ORR and PFS. mut, mutation(s); n, number of study participants or samples in the indicated subgroup

To determine whether mutation clonality influences clinical outcomes in patients with ER+/HER2– mBC, we analyzed objective response rates (ORR) and progression-free survival (PFS) of the SANDPIPER participants against treatment regimen (i.e., pbo + fulv or tas + fulv) and against clonal or subclonal status in both the multiple and single *PIK3CA*mut cohorts. In the multiple *PIK3CA*mut cohort (*n* = 66), a higher ORR was observed in patients whose samples were categorized as clonal multiple *PIK3CA*mut [30% (95% CI, 17–45)] vs those whose samples were categorized as subclonal multiple *PIK3CA*mut [5.3% (95% CI, 0.13–26)], regardless of treatment regimen (*p* = 0.021) (Fig. 4A). Within the treatment arms, this finding was not significant for patients treated specifically with pbo + fulv [11% and 0.00% in the clonal vs subclonal subgroups, respectively (95% CI, 1.4–35 vs 0.00–52), *p* = 1.0] (Fig. 4B). However, this finding was significant for patients treated with tas + fulv [41% and 7.1% in the clonal vs subclonal subgroups, respectively (95% CI, 24–61 vs 0.18–34), *p* = 0.033] (Fig. 4C).

In comparing patient ORR for samples of single *PIK3CA*mut clonality status, ORR did not significantly differ in the total analyzed population [either treatment regimen, clonal vs subclonal single *PIK3CA*mut status: 15% vs 25% (95% CI, 11–20 vs 9.8–47), *p* = 0.24] or under either SANDPIPER treatment regimen [pbo + fulv, clonal vs subclonal status: 9.5% vs 17% (95% CI, 3.9–19 vs 0.42–64), *p* = 0.48; tas + fulv, clonal vs subclonal status: 17% vs 28% (95% CI 12–24 vs 9.7–53), *p* = 0.33]. Numerically higher ORR was seen in patients with ctDNA categorized as subclonal single *PIK3CA*mut vs clonal single *PIK3CA*mut, but did not reach significance (Additional File 1: Fig. S4A-SC). This may suggest that subclonal single *PIK3CA*mut tumors are less aggressive than clonal single *PIK3CA*mut tumors, however, further investigation and a larger sample size are warranted to confirm this observation and to better elucidate the potential associated biological underpinnings.

When we examined PFS within each SANDPIPER treatment arm, the results were complementary to the ORR analysis. Specifically, median PFS (mPFS) was longer for patients whose baseline ctDNA samples were categorized as clonal multiple *PIK3CA*mut vs subclonal multiple *PIK3CA*mut for both the pbo + fulv arm [mPFS = 3.9 vs 2.0 months, respectively; Hazard Ratio (HR) = 0.19 (95% CI, 0.051–0.73), *p* = 0.025] and the tas + fulv arm [mPFS = 7.6 vs 5.1 months, respectively; HR = 0.37 (95% CI, 0.16–0.87), *p* = 0.027] (Fig. 4D). mPFS was longest overall for patients who received tas + fulv and whose baseline ctDNA harbored clonal multiple *PIK3CA*mut (Fig. 4D). For patients whose baseline ctDNA harbored only a single *PIK3CA*mut, we observed no significant difference in mPFS between clonality subgroups under either treatment arm (Additional file 1: Fig. S4D). Lastly, the ORR and mPFS of the subclonal multiple *PIK3CA*mut subgroup wherein one of the *PIK3CA* mutations has a clonality estimate of clonal (*n* = 15) were similar to the clinical outcomes observed in the entire subclonal multiple *PIK3CA*mut cohort (data not shown).

## Discussion

As previously described, tumor cells harboring multiple *PIK3CA*mut occurring *in cis* confer increased sensitivity to p110α inhibition compared to tumor cells harboring only a single *PIK3CA*mut or multiple *PIK3CA*mut occurring *in trans* [16]. Therein, the authors performed both long-range single-molecule real-time sequencing (SMRTseq) [26] of circular DNA templates from multiple *PIK3CA*mut breast cancer cell lines and a limited number of fresh tumor samples, as well as an in silico clonality estimation analysis [27] on a previously-published breast cancer sequencing dataset [6] to infer *in cis* vs *in trans* status of multiple *PIK3CA*mut. For the latter, co-occurring clonal *PIK3CA*mut were representative of multiple *PIK3CA*mut *in cis*, whereas a mix of co-occurring clonal and subclonal, or subclonal only, *PIK3CA*mut were representative of multiple *PIK3CA*mut *in trans*. Together, these efforts revealed that multiple *PIK3CA*mut identified in breast cancers more commonly occur *in cis* [16].

We have recently shown that clonal multiple *PIK3CA*mut predominantly occur *in cis* while subclonal multiple *PIK3CA*mut frequently occur *in trans* [25]. Based on this, we were able to confirm the preclinical findings from Vasan, et al. [16] in a phase III clinico-genomics dataset. Although some of the analysis subgroups were relatively small, we nevertheless observed prolonged PFS and increased ORR for patients with ctDNA harboring clonal vs subclonal multiple *PIK3CA*mut, which was enhanced for those patients randomized to treatment with taselisib + fulvestrant. This is analogous to the preclinical findings that multiple *PIK3CA*mut breast epithelial cells are more sensitive to alpelisib treatment when the *PIK3CA* mutations are *in cis* compared to when they are *in trans* [16]. These trends were also observed in a phase I/Ib population of patients with HR+/HER2– mBC treated with inavolisib (GDC-0077) alone or with endocrine therapy and with or without palbociclib (NCT03006172). Specifically, the fraction of patients who experienced partial response or stable disease in this study was higher in those patients from whom multiple *PIK3CA*mut were detected in baseline ctDNA compared to those in whom only a single *PIK3CA*mut was detected [28]. Furthermore, from a case report of a patient with ER+/HER2– mBC treated with alpelisib plus fulvestrant who achieved a partial response, molecular profiling revealed that both the primary tumor and metastatic lesions harbored multiple *PIK3CA*mut [29].

**Fig. 5:**
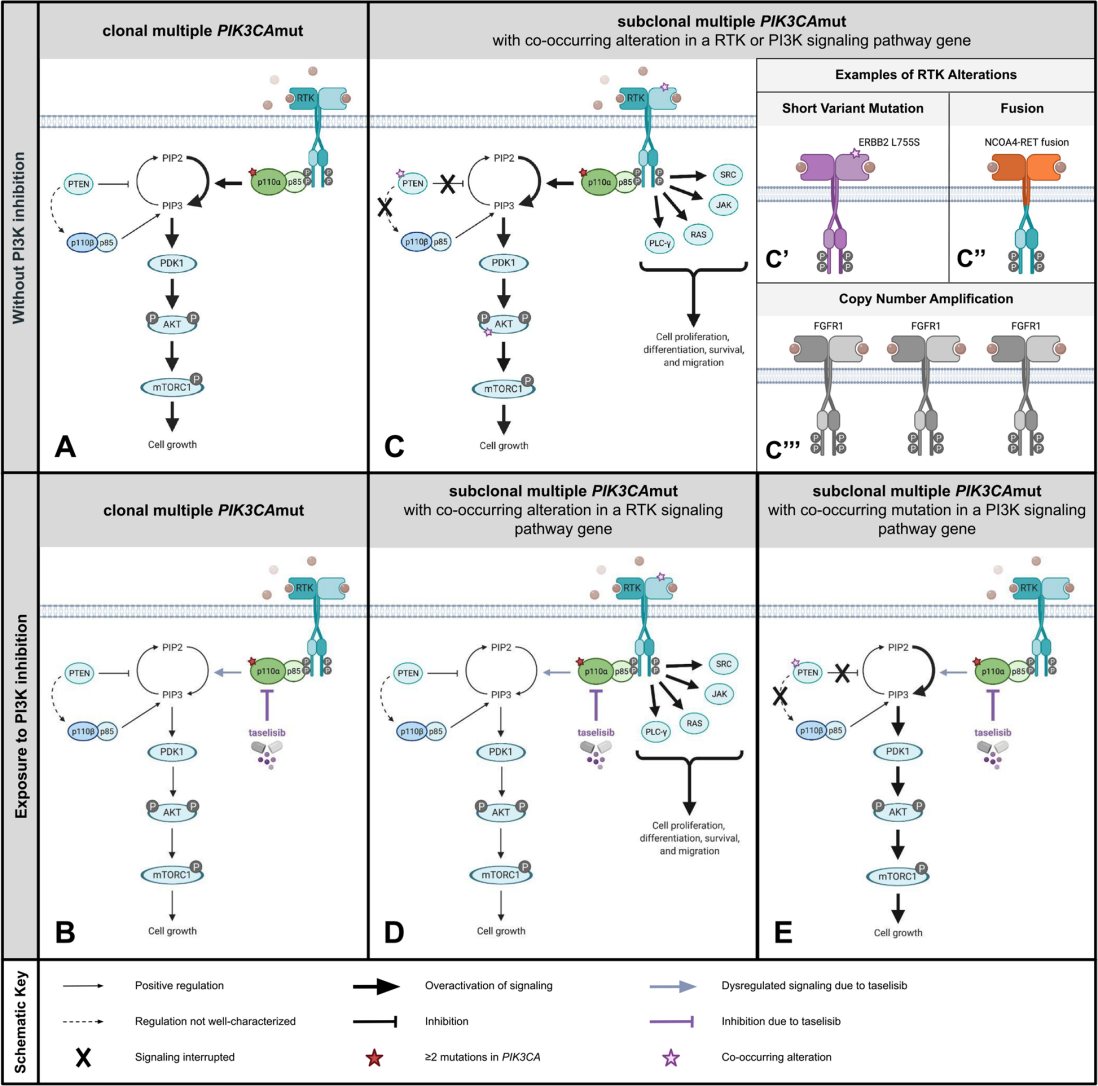
Models for interpreting predicted reliance on PI3K pathway signaling and sensitivity to p110α inhibition. **A** Tumors with clonal multiple *PIK3CA*mut are sufficient to drive tumor growth and proliferation through hyperactivation of the PI3K pathway alone and (**B**) are highly sensitive to PI3K inhibition. **C** Tumors with subclonal multiple *PIK3CA*mut may be insufficient to adequately drive growth and proliferation alone and may have co-occurring alterations in RTK and/or non-*PIK3CA* PI3K pathway genes (C’, C’’, C’’’). In these instances, tumor growth and proliferation may not be fully abrogated by PI3K inhibition alone due to (**D**) activation of additional parallel signaling pathways, and/or (**E**) further enhanced PI3K/AKT pathway signaling

Supportive of the correlations we observed with clinical response, our analysis of multiple *PIK3CA*mut clonality against the broader genomic landscape of breast cancer ctDNA reveals an updated model for dependence on the PI3K pathway. To illustrate the interpretation of our findings in conjunction with previously published work, we have generated the model shown in Fig. 5. We hypothesize clonal multiple *PIK3CA*mut alone may be sufficient to drive tumor growth and proliferation through hyperactivation of the PI3K/AKT pathway (Fig. 5A) and as a result, are highly sensitive to PI3K inhibition (Fig. 5C). Conversely, our data also suggest subclonal multiple *PIK3CA*mut may require additional input from co-altered signaling pathway genes, including alterations in RTK pathway genes, to fully drive tumor growth and proliferation through the PI3K and parallel pathways (Fig. 5B). As such, tumor growth and proliferation may not be fully abrogated by PI3K inhibition alone due to potential bypass mechanisms. Ultimately, our observations suggest that rational therapeutic combinations should be considered – particularly for those patients with tumors harboring subclonal multiple *PIK3CA*mut, wherein co-targeting of concomitantly dysregulated RTK or non-*PIK3CA* PI3K pathway genes in addition to p110α may be beneficial (Fig. 5D-E).

An open question, however, is whether the clonality of *PIK3CA*mut – especially those of subclonal status – is representative of co-occurrence or mutual exclusivity of alterations in RTK and PI3K pathway genes at the single-cell level. If *PIK3CA*mut and RTK/PI3K pathway alterations occur in separate cells, a treatment combination may provide more benefit than if these alterations were to occur in the same cell. Although we are likely a distant future away from integrating single-cell technologies into clinical practice, which may afford an opportunity to better characterize and refine our understanding of the clonality of alterations in key signaling pathways.

Our study also raises questions about the importance of multiple *PIK3CA*mut across cancer types. First and foremost, do our findings in HR+/HER2– metastatic breast cancer translate to other cancer types harboring multiple *PIK3CA*mut? One could surmise that other cancer types exhibit different levels of dependence on the PI3K/AKT pathway compared to their reliance on other cancer-dysregulated signaling pathways, and depending on the genomic background of those cancers, they may be differentially influenced by the presence of multiple *PIK3CA*mut. According to the analysis by Vasan, et al., colorectal (CRC) and uterine/endometrial (UC/EC) cancers harbor the next highest proportion of multiple *PIK3CA*mut after breast cancers [16]. Through the independent tumor tissue database, we analyzed 635 CRC and 681 UC/EC tumor tissue samples with clonal vs subclonal multiple *PIK3CA*mut against the potential for co-occurring RTK and non-*PIK3CA* PI3K pathway alterations and observed no differences (data not shown); as alluded to, this may be due to inherent differences in the biologies of these tumor types compared to breast cancer and/or due to an underpowered dataset. To address exactly those questions about the importance of multiple *PIK3CA*mut beyond breast cancer, new clinical studies are underway. One such study is the ongoing TAPISTRY (Tumor-Agnostic Precision Immuno-Oncology and Somatic Targeting Rational for You) platform study (NCT04589845). Leveraging site-based NGS results to augment enrollment, TAPISTRY will assess the efficacy of the p110α inhibitor, inavolisib, in adolescent and adult patients with a range of advanced or metastatic tumor types harboring multiple *PIK3CA*mut in patients that have failed conventional therapeutic options.

## Conclusions

In summary, our study establishes the prospect that multiple clonal *PIK3CA*mut are prognostic of response to PI3K inhibition. These data provide additional rationale for the clinical investigation of the clonality status of *PIK3CA*mut with PI3K-inhibitors like the p110α-isoform selective inhibitor, inavolisib, alone or in combination with other rationally selected therapies.

## Supplementary Information

**Additional file 1:** PDF file, containing the article-associated supplementary tables and figures. **Table S1.** Clonality subgroup nomenclature and associated definitions for single and multiple PIK3CAPIK3CAmut; **Table S2.** Clonality estimation and tabulation of multiple PIK3CAPIK3CAmut identified in baseline ctDNA from SANDPIPER participants; **Table S3.** Clonality estimation and tabulation of single PIK3CAPIK3CAmut identified in baseline ctDNA from SANDPIPER participants; **Table S4.** Signaling pathway gene lists utilized in pathway-level analyses; **Table S5.** Fraction of samples with altered signaling pathway genes between SANDPIPER treatment groups; **Table S6.** Fraction of samples with altered signaling pathway genes between SANDPIPER treatment groups stratified by clonality status; **Fig. S1.** Gene alteration rates between SANDPIPER baseline ctDNA samples categorized as clonal vs subclonal single PIK3CAPIK3CAmut; **Fig. S2.** Pathway-level co-alteration analyses between ctDNA samples harboring clonal vs subclonal single PIK3CAPIK3CAmut; **Fig. S3.** Consort diagram for the clonality analysis of an independent breast tumor tissue dataset; **Fig. S4.** ORR and PFS of SANDPIPER participants whose baseline ctDNA samples harbored single PIK3CAPIK3CAmut. 

## Data Availability

All relevant data are provided as supplementary information. Information on the availability of data from the SANDPIPER clinical trial can be found in the published primary clinical manuscript [14]. In brief, qualified researchers can request access to individual patient-level data through the clinical study data request platform (https://vivli.org/). SANDPIPER trial participants did not give consent to publicly share their individualized comprehensive genomic profiling data. However, the clinical study data (https://vivli.org/) and requests for genomic profiling data and analyses can be released to qualified requestors along with necessary agreements to enforce terms such as security, patient privacy, and consent of specified data use, consistent with evolving and applicable data protection laws. For up-to-date details on Roche's Global Policy on the Sharing of Clinical Information and how to request access to related clinical study documents, see here: https://go.roche.com/data_sharing. Due to HIPAA requirements, Foundation Medicine, Inc. (FMI) is not consented to share individualized patient genomic data, which contains potentially identifying or sensitive information. Foundation Medicine is committed to collaborative data analysis and has well established and widely used mechanisms by which qualified researchers can query our genomic database of > 600,000 de-identified sequenced cancers. More information and mechanisms for data access can be obtained by contacting the corresponding author or the Foundation Medicine Data Governance Council at data.governance.council@foundationmedicine.com. This study made use of publicly available packages with R version 3.6.1 and Python 2.7. Code used to generate figures are available upon request.

## Abbreviations

2L: Second-line therapy
AF: Allele fraction or allele frequency
AKT: Protein kinase B
BICR-PFS: Progression-free survival (PFS) per blinded independent central review
BC(s): Breast cancer(s)
BOR: Best objective response
*BRCA2*: BReast CAncer gene 2
CAP: College of American Pathologists
CBR: Clinical benefit rate
CGP: Comprehensive genomic profiling
CI: Confidence interval
CLIA: Clinical Laboratory Improvement Amendments
CR: Complete response
CRC: Colorectal
ctDNA: Circulating tumor DNA
DNA: Deoxyribonucleic acid
DoR: Duration of objective response
EC: Endometrial cancer
ER+: Estrogen receptor-positive
*ERBB2*: Erb-b2 receptor tyrosine kinase 2
F1: FoundationOne^®^
F1CDx: FoundationOne^®^CDx
F1L: FoundationOne^®^Liquid assay
FDA: Food and Drug Administration
FFPE: Formalin-fixed, paraffin-embedded
FMI: Foundation Medicine, Inc.
fulv: Fulvestrant
HER2–: Human epidermal growth factor receptor 2-negative
HER2+: Human epidermal growth factor receptor 2-positive
HR+: Hormone receptor-positive
IRB: Institutional review board
MAPK: Mitogen activated protein kinase (pathway)
mc: Mutated copies
*MDM2*: Murine double minute 2 gene
MND: Mutation not detected
mPFS: Median progression-free survival
MSAF: Maximum somatic allele fraction
mTOR: Mammalian target of rapamycin
mut: Mutated, mutation(s), or mutant
NGS: Next-generation sequencing
NMD: No mutation detected
ORR: Objective response rate
OS: Overall survival
p110α: Phosphatidylinositol-4,5-bisphosphtate 3-kinase catalytic subunit alpha protein, 110 kilodaltons
p110β: Phosphatidylinositol-4,5-bisphosphtate 3-kinase catalytic subunit beta protein, 110 kilodaltons
p53: Transformation-related protein 53
PFS: Progression-free survival
PI3K: Phosphatidylinositol 3-kinase complex
PI3Kα: Phosphoinositol 3-kinase alpha
*PIK3CA*: Phosphatidylinositol-4,5-bisphosphtate 3-kinase catalytic subunit alpha gene
*PIK3CA*mut: *PIK3CA* mutated, mutation(s), or mutant
pbo: Placebo
PR: Partial response
RECIST: Response Evaluation Criteria in Solid Tumors
RTK: Receptor tyrosine kinase
SGZ: Somatic-germline-zygosity
SMRTseq: Single-molecule real-time sequencing
SNV: Single nucleotide variant
TAPISTRY: Tumor-Agnostic Precision Immuno-Oncology and Somatic Targeting Rational for You platform study
tas: Taselisib
UC: Uterine cancer
VAF: Variant allele fraction
wc: Wild-type copies
