# Supplementary material for: Multiple PIK3CA mutation clonality correlates with outcomes in taselisib + fulvestrant-treated ER+/HER2–, PIK3CA-mutated breast cancers
Source: Genome Med. 2023 Apr 26;15:28. doi: 10.1186/s13073-023-01181-8 (PMC10131374; doi:10.1186/s13073-023-01181-8)
Supplement: Supplementary file 1 — Additional file 1: PDF file, containing the article-associated supplementary tables and figures. Table S1. Clonality subgroup nomenclature and associated definitions for single and multiple PIK3CAPIK3CAmut; Table S2. Clonality estimation and tabulation of multiple PIK3CAPIK3CAmut identified in baseline ctDNA from SANDPIPER participants; Table S3. Clonality estimation and tabulation of single PIK3CAPIK3CAmut identified in baseline ctDNA from SANDPIPER participants; Table S4. Signaling pathway gene lists utilized in pathway-level analyses; Table S5. Fraction of samples with altered signaling pathway genes between SANDPIPER treatment groups; Table S6. Fraction of samples with altered signaling pathway genes between SANDPIPER treatment groups stratified by clonality status; Fig. S1. Gene alteration rates between SANDPIPER baseline ctDNA samples categorized as clonal vs subclonal single PIK3CAPIK3CAmut; Fig. S2. Pathway-level co-alteration analyses between ctDNA samples harboring clonal vs subclonal single PIK3CAPIK3CAmut; Fig. S3. Consort diagram for the clonality analysis of an independent breast tumor tissue dataset; Fig. S4. ORR and PFS of SANDPIPER participants whose baseline ctDNA samples harbored single PIK3CAPIK3CAmut. [file 13073_2023_1181_MOESM1_ESM.pdf]

SUPPLEMENTARY TABLES AND FIGURES FOR:

Multiple *PIK3CA* mutation clonality correlates with outcomes in taselisib + fulvestrant-treated ER+/HER2–, *PIK3CA*-mutated breast cancers

Katherine E. Hutchinson[1]*, Jessica W. Chen[1]*, Heidi M. Savage[1], Thomas J. Stout[2], Frauke Schimmoller[2], Javier Cortés[3], Susan Dent[4], Nadia Harbeck[5], William Jacot[6], Ian Krop[7], Sally E. Trabucco[8], Smruthy Sivakumar[8], Ethan S. Sokol[8], Timothy R. Wilson[1]

AFFILIATIONS

*equal contributions

[1] Oncology Biomarker Development, Genentech, Inc., South San Francisco, CA, USA

[2] Product Development Clinical Oncology, Genentech, Inc., South San Francisco, CA, USA

[3] International Breast Cancer Center (IBCC), Quironsalud Group, Madrid & Barcelona, Spain; Vall d'Hebron Institute of Oncology (VHIO), Barcelona, Spain

[4] Duke Cancer Institute, Duke University, Durham, NC, USA

[5] Breast Center, Department Gynecology and Obstetrics and Comprehensive Cancer Center (CCC) Munich, Ludwig Maximilians University (LMU) Hospital, Munich, Germany

[6] Institut du Cancer de Montpellier (ICM) Val d'Aurelle, Montpellier University, Montpellier, France

[7] Yale Cancer Center, New Haven, CT, USA

[8] Foundation Medicine, Inc., Cambridge, MA, USA

CORRESPONDING AUTHOR

Timothy R. Wilson

1 DNA Way

South San Francisco, CA 94080

Phone: +1 (650) 225-1000

Email: wilson.timothy@gene.com

SUPPLEMENTARY TABLES

**Table S1. Clonality subgroup nomenclature and associated definitions for single and multiple *PIK3CA*mut.**	
**Clonality Category**	**Definition**
clonal single *PIK3CA*mut	A single *PIK3CA* mutation was detected in the sample with a clonality estimation of clonal.
subclonal single *PIK3CA*mut	A single *PIK3CA* mutation was detected in the sample with a clonality estimation of subclonal.
clonal multiple *PIK3CA*mut	≥2 *PIK3CA* mutations were detected in the sample and ≥2 of those *PIK3CA* mutations have a clonality estimation of clonal.
subclonal multiple *PIK3CA*mut	≥2 *PIK3CA* mutations were detected in the sample, but ≤1 of those *PIK3CA* mutations has a clonality estimation of clonal.
In this analysis, *PIK3CA* mutation is defined as a pathogenic single nucleotide variant (SNV).	

**Table S2. Clonality estimation and tabulation of multiple *PIK3CA*mut identified in baseline ctDNA from SANDPIPER participants.**			
**multiple *PIK3CA*mut (estimated clonality[a])**		**pbo+fulv arm**	**tas+fulv arm**
H1047R (clonal)	E542Q (clonal)	.	2
	E39K (clonal)	.	1
	E418K (clonal)	.	1
	E726K (clonal)	.	1
	N1044S (clonal)	.	1
	N345K (clonal)	.	1
	Q546E (clonal)	.	1
	Q546H (clonal)	.	1
	E542K (clonal)	1	.
	E81K (clonal)	1	.
	Q546P (clonal)	1	.
	R108H (clonal)	1	.
	R88Q (clonal)	1	.
	E726K (subclonal)	.	3
	R115L (subclonal)	.	1

	R88Q (subclonal)	.	1
	R93W (subclonal)	.	1
	E542K, E545K, E545Q, E726K, E81K, Q75E, R93Q (all subclonal)	.	1
H1047L (clonal)	E365K (clonal)	.	2
	E453K (clonal)	.	1
	N345K (clonal)	.	1
	E418K (clonal)	1	.
	E542K, E726K (all subclonal)	1	.
	E542K, E726K, E453K, K111N (all subclonal)	.	1
H1047Y (clonal)	E545K (clonal)	.	1
E545K (clonal)	E726K (clonal)	2	5
	E453K (clonal)	2	.
	E453Q (clonal)	.	1
	M1004I (clonal)	.	1
	P539R (clonal)	.	1
	E726K (subclonal)	1	1
E545G (clonal)	E39K (clonal)	1	.
E542K (clonal)	E365K, C420R (all clonal)	.	1
	E418K (clonal)	.	1
	M1043I (clonal)	.	1
	E453K (clonal)	1	.
	E726K (clonal)	1	.
	E453Q (clonal), E726K (subclonal)	.	1
	E726K (clonal), H1047R (subclonal), E545K (subclonal), E453Q (subclonal), M1043I (subclonal)	.	1
	R108H (subclonal)	.	1
	Y1021H (subclonal)	1	.
N345K (clonal)	I1058L (clonal)	.	1
	G1049R (clonal)	1	.
	G1049S (clonal)	1	.
	T1025A (clonal)	1	.

C420R (clonal)	G1049R (clonal)	1	.
	E726K, E365K (all subclonal)	1	.
	E726K (subclonal)	.	1
Q546R (clonal)	E453K (clonal), E453Q (subclonal)	1	.
E545A (subclonal)	E726K (clonal)	.	1
E545K (subclonal)	E542K (subclonal)	.	2
	E542Q (subclonal)	.	1
H1047R (subclonal)	N345K (subclonal)	1	.

[a] Light gray-shaded rows denote multiple *PIK3CA*mut detected in patient baseline ctDNA categorized as "subclonal multiple *PIK3CA*mut" (refer to **Table S1** for definitions).
Lack of a numeric value is denoted by "." and indicates that no sample in the respective treatment arm harbored the specified combination of *PIK3CA*mut.
pbo, placebo; fulv, fulvestrant; N, sample size; tas, taselisib

Table S3. Clonality estimation and tabulation of single *PIK3CA*mut identified in baseline ctDNA from SANDPIPER participants.

p110α mutation	pbo + fulv			tas + fulv		
	single *PIK3CA*mut (TOTAL)	clonal *PIK3CA*mut	subclonal *PIK3CA*mut	single *PIK3CA*mut (TOTAL)	clonal *PIK3CA*mut	subclonal *PIK3CA*mut
H1047R	28	28	.	72	64	8
H1047L	3	3	.	12	10	2
E545K	28	24	4	51	49	2
E545A	.	.	.	3	3	.
E545G	.	.	.	1	1	.
E542K	7	7	.	28	23	5
E542A	.	.	.	1	1	.
N345K	6	5	1	11	11	.
C420R	2	2	.	4	4	.
G1049R	2	2	.	3	3	.
Q546R	2	2	.	1	1	.
Q546K	1	1	.	2	2	.

M1043I	1	.	1	.	.	.
G106V	.	.	.	1	1	.
R108H	.	.	.	1	.	1

Lack of a numeric value is denoted by "." and indicates that no sample in the respective treatment arm harbored the specified *PIK3CA*mut.
pbo, placebo; fulv, fulvestrant; tas, taselisib

Table S4. Signaling pathway gene lists utilized in pathway-level analyses.

Pathway	Gene List[a]
**PI3K** (exclusive of *PIK3CA*)	*AKT1, MTOR, PTEN, STK11*
**p53**	*ATM, BRCA1, CDK12, CDKN2A, CHEK2, MDM2, NPM1, TP53*
**RTK**	*ALK, EGFR, ERBB2, FGFR1/2/3, KIT, MET, PDGFRA/B, RET, ROS1*
**MAPK**	*ARAF, BRAF, HRAS, JAK2, KRAS, MAP2K1/2, NF1, NRAS, RAF1*

[a]Only genes covered in the FoundationOne® Liquid (F1L) assay were included.
MAPK, mitogen-activated protein kinase; PI3K, phosphoinositide 3-kinase; RTK, receptor tyrosine kinase

Table S5. Fraction of samples with altered signaling pathway genes between SANDPIPER treatment groups

Signaling Pathway	pbo +fulv n of 43 (%)	tas + fulv n of 23 (%)	p-value Fisher's Exact Test	BH-adjusted p-value
**RTK**	10 (23)	8 (35)	0.39	0.52
**MAPK**	9 (21)	6 (26)	0.76	0.76
**PI3K**	5 (12)	6 (26)	0.17	0.52
**p53**	21 (49)	15 (65)	0.30	0.52

BH, Benjamini-Hochberg; MAPK, mitogen-activated protein kinase; PI3K, phosphoinositide 3-kinase; RTK, receptor tyrosine kinase; pbo, placebo; fulv, fulvestrant; tas, taselisib; n, number of altered samples

**Table S6. Fraction of samples with altered signaling pathway genes between SANDPIPER treatment groups stratified by clonality status**					
	**Signaling Pathway**	**pbo + fulv n of 29 (%)**	**tas + fulv n of 18 (%)**	**p-value Fisher's Exact Test**	**BH-adjusted p-value**
**Clonal multiple *PIK3CA*mut**	**RTK**	5 (17)	4 (22)	0.72	0.96
	**MAPK**	7 (24)	6 (33)	0.52	0.83
	**PI3K**	2 (6.9)	3 (17)	0.36	0.72
	**p53**	12 (41)	12 (67)	0.14	0.56
	**Signaling Pathway**	**pbo + fulv n of 14 (%)**	**tas + fulv n of 5 (%)**	**p-value Fisher's Exact Test**	**BH-adjusted p-value**
**Subclonal multiple *PIK3CA*mut**	**RTK**	5 (36)	4 (80)	0.14	0.56
	**MAPK**	2 (14)	0 (0.00)	1.0	1.0
	**PI3K**	3 (21)	3 (60)	0.26	0.69
	**p53**	9 (64)	3 (60)	1.0	1.0

BH, Benjamini-Hochberg; MAPK, mitogen-activated protein kinase; PI3K, phosphoinositide 3-kinase; RTK, receptor tyrosine kinase; pbo, placebo; fulv, fulvestrant; tas, taselisib; n, number of altered samples

SUPPLEMENTARY FIGURES & LEGENDS

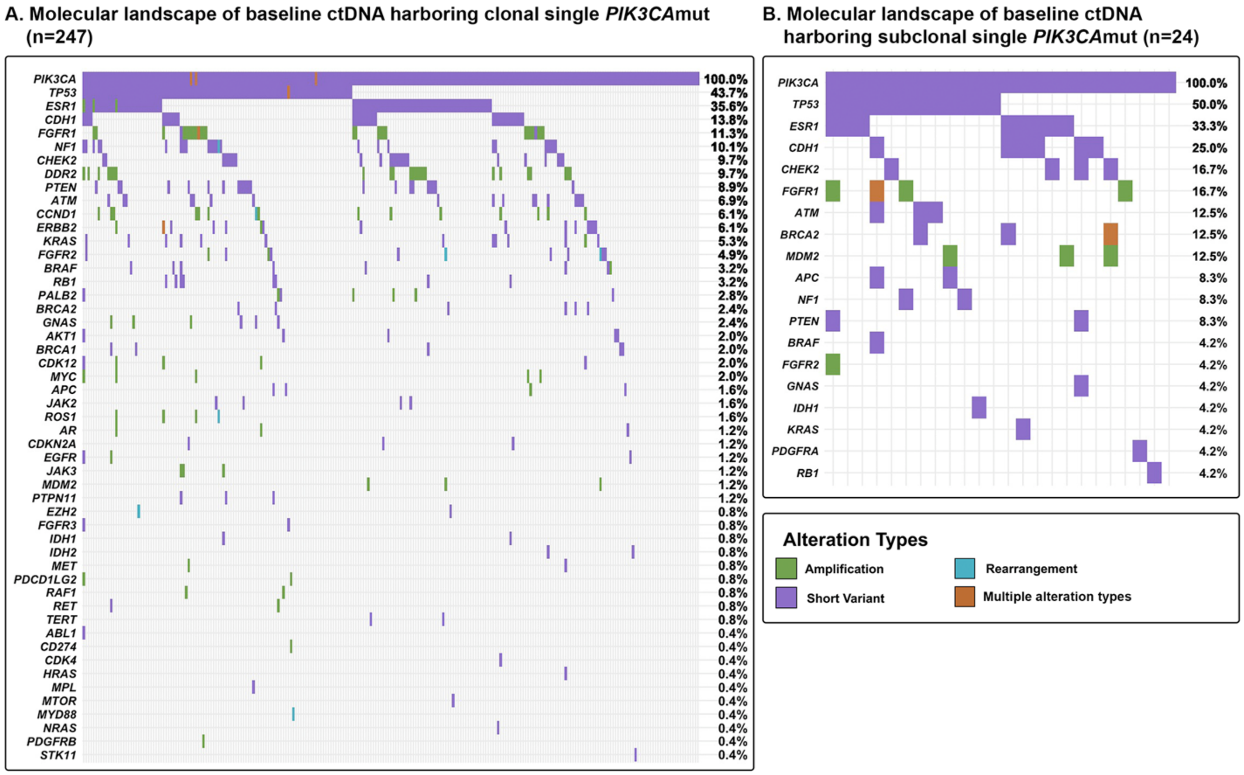

Fig. S1. Gene alteration rates between SANDPIPER baseline ctDNA samples categorized as clonal vs subclonal single *PIK3CA*mut. Tile plots from FoundationOne® Liquid (F1L) sequencing of baseline ctDNA from patients enrolled to SANDPIPER exhibit the somatic alterations co-occurrent with samples categorized as **(A)** clonal single *PIK3CA*mut or **(B)** subclonal single *PIK3CA*mut. Samples are represented in the columns, whereas genes are represented in the rows.

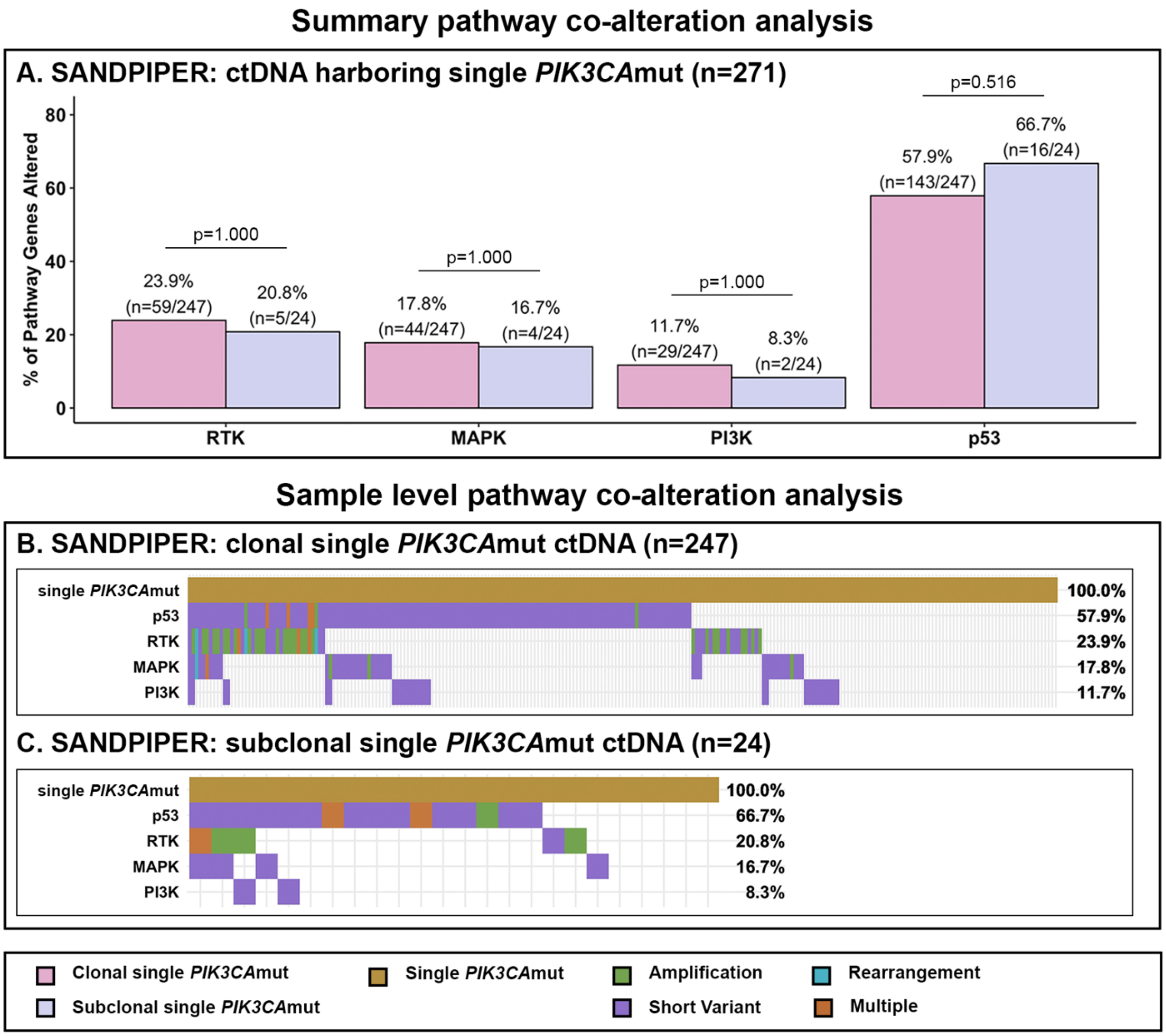

Fig. S2. Pathway-level co-alteration analyses between ctDNA samples harboring clonal vs subclonal single *PIK3CA*mut. **(A)** Summary diagram of pathway-level alteration rates in baseline ctDNA from patients enrolled to SANDPIPER. **(B)** Sample-level tile plot of analysis in **(A)** for samples categorized as clonal single *PIK3CA*mut. **(C)** Sample-level tile plot of analysis in **(A)** for samples categorized as subclonal single *PIK3CA*mut.

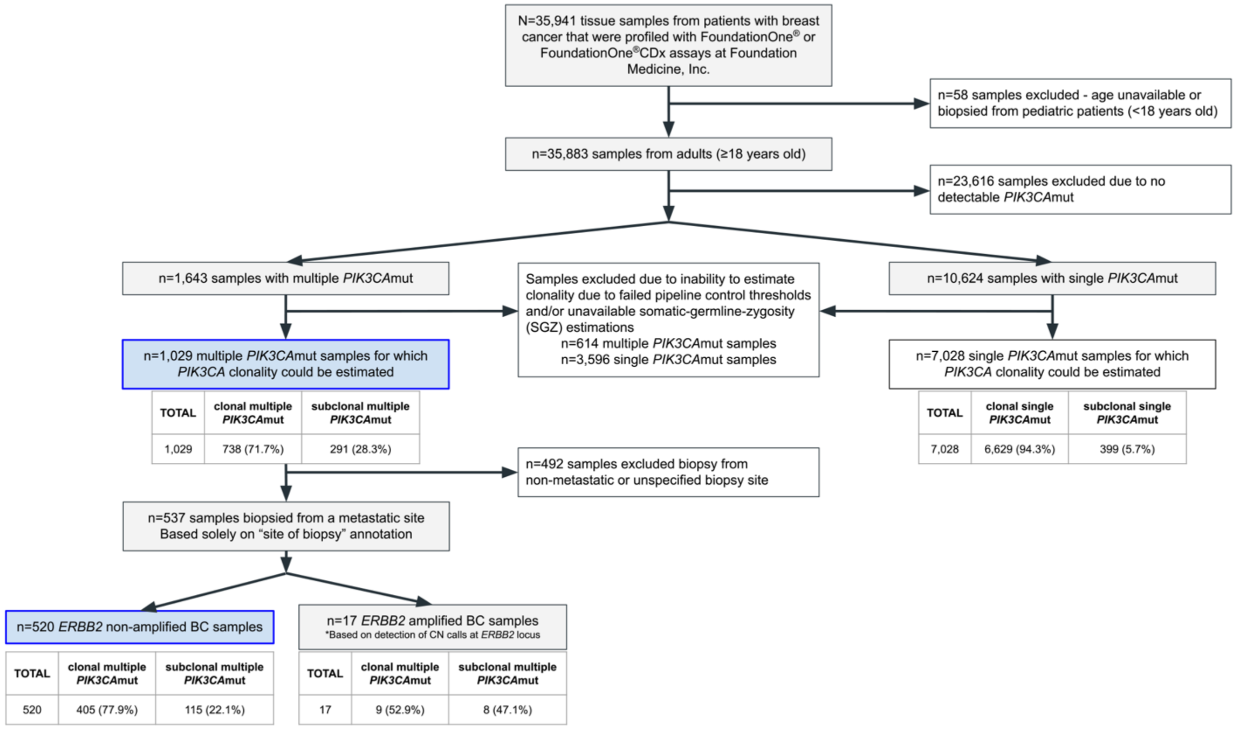

Fig. S3. Consort diagram for the clonality analysis of an independent breast tumor tissue dataset.

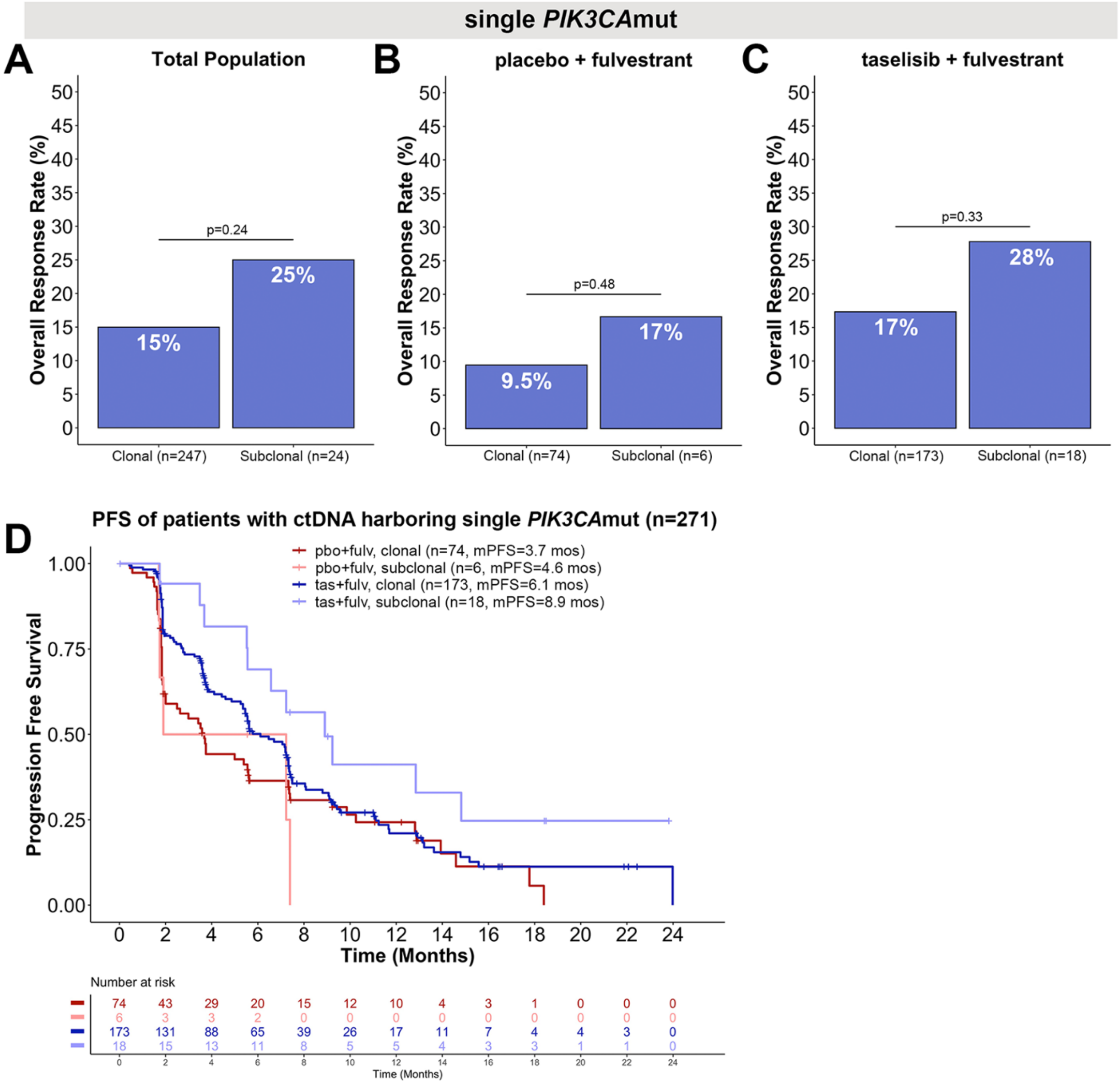

Fig. S4. ORR and PFS of SANDPIPER participants whose baseline ctDNA samples harbored single *PIK3CA*mut. **(A - C)** Bar plots of overall objective response rates (ORR) of SANDPIPER study participants compared between those whose baseline ctDNA harbored clonal vs subclonal single *PIK3CA*mut. **(A)** Patients who received either study treatment regimen: clonal [37 responses of 247 participants = 15% ORR (95% CI, 11-20)] vs subclonal [6 responses of 24 participants = 25% ORR (95% CI, 9.7-47)]; *p*=0.24. **(B)** Patients who received placebo + fulvestrant: clonal [7 responses of 74 participants = 9.5% ORR (95% CI, 3.9-19)] vs subclonal [1 response of 6 participants = 17% ORR (95% CI, 0.42-64)];

p=0.48. **(C)** Patients who received taselisib + fulvestrant: clonal [30 responses of 173 participants = 17% ORR (95% CI, 12-24)] vs subclonal [5 responses of 18 participants = 28% ORR (95% CI, 9.7-54)]; *p*=0.33. **(D)** Shown by Kaplan-Meier curves, median progression-free surivval (PFS) was no different for patients whose corresponding baseline ctDNA harbored clonal single *PIK3CA*mut vs subclonal single *PIK3CA*mut and were treated with either placebo + fulvestrant [mPFS = 3.7 vs 4.6 months, respectively; Hazard Ratio (HR) = 0.77 (95% CI, 0.31-1.9), p=0.59] or with taselisib + fulvestrant [mPFS = 6.1 vs 8.9 months, respectively; HR = 1.6 (95% CI, 0.88-3.0), *p*=0.096]. See Methods for details regarding calculations and statistics for ORR and PFS. mut, mutation(s); n, number of participants in the indicated subgroup.
